# Bilateral ovarian metastases from gastric signet-ring cell carcinoma in an 18-year-old: a case report and narrative review

**DOI:** 10.3389/fonc.2026.1744052

**Published:** 2026-02-24

**Authors:** Andreea Boiangiu, Liviu Bilteanu, Andreea Costeschi, Catalina-Andreea Nicolae, Romina-Marina Sima, Radu Vladareanu, Andreea-Iren Serban, Serban-Andrei Marinescu, Valentin-Nicolae Varlas, Cezar Iliescu, Alexandru Filipescu

**Affiliations:** 1 Carol Davila University of Medicine and Pharmacy, Bucharest, Romania; 2 Elias Emergency University Hospital, Bucharest, Romania; 3 Faculty of Biology, University of Bucharest, Bucharest, Romania; 4 Laboratory for Molecular Nanotechnologies, National Institute for Research and Development in Microtechnologies - IMT Bucharest, Voluntari, Romania; 5 Department of Preclinical Sciences, Faculty of Veterinary Medicine, University of Agronomic Sciences and Veterinary Medicine, Bucharest, Romania; 6 Division of Internal Medicine, The University of Texas MD Anderson Cancer Center, Houston, TX, United States

**Keywords:** adolescent, bilateral ovarian masses, gastric signet-ring cell carcinoma, Krukenberg Tumor, ovarian metastasis

## Abstract

**Background/objectives:**

Krukenberg tumors (KTs) are rare metastatic ovarian neoplasms, most frequently secondary to gastric adenocarcinomas, and typically occur in women aged 30–60 years. Their presentation in adolescents is exceedingly uncommon and often leads to diagnostic delays due to nonspecific symptoms and low clinical suspicion.

**Case presentation:**

We describe the case of an 18-year-old female presenting with progressive abdominal pain and bilateral adnexal masses. Imaging suggested advanced ovarian pathology, and surgical exploration with histopathological evaluation confirmed metastatic signet-ring cell carcinoma. Upper gastrointestinal endoscopy subsequently identified a poorly differentiated gastric adenocarcinoma, establishing the diagnosis of KT.

**Methods:**

To place this case in context, a narrative literature review was performed using PubMed, Google Scholar, and ScienceDirect. English-language case reports and case series describing KTs in patients aged ≤20 years were selected. Fifteen studies were analyzed regarding clinical presentation, histopathological features, diagnostic approach, and patient outcomes.

**Results:**

The majority of reported cases demonstrated bilateral ovarian involvement, vague abdominal symptoms, and gastric origin. Diagnostic delays were frequent, and prognosis remained poor despite multimodal treatment strategies.

**Conclusions:**

This case highlights the importance of considering metastatic gastrointestinal malignancies in the differential diagnosis of ovarian tumors in adolescents and emphasizes the value of early endoscopic assessment. By combining a rare adolescent case with a focused literature review, this work provides one of the few comprehensive syntheses of KTs in patients under 20 years, offering a unique contribution to improving clinical awareness and guiding future diagnostic strategies.

## 1 Introduction

Krukenberg tumors (KTs) represent a rare subtype of metastatic ovarian neoplasms, characterized histologically by mucin-producing signet ring cells. They account for between 1% and 2% of all ovarian cancers. They are estimated to represent approximately 30–40% of all secondary ovarian metastases (1). The KTs frequency varies with the prevalence of gastric carcinoma in the studied population; for example, in Japan, they account for a significant proportion (17.8%) of all ovarian cancers (2, 3). It is important to note that adenocarcinomas composed of signet ring cells from various organs tend to metastasize to the ovaries much more commonly than adenocarcinomas of other histological types from the same primary sites (2).

Although Friedrich Krukenberg described the tumors in 1896, he erroneously interpreted them as primary ovarian fibro-sarcomas containing mucinous cells. Their true metastatic nature was established six years later by Schlagenhaufer (2). The stomach constitutes the most prevalent primary site, followed by malignancies originating from the colon, appendix, and breast, especially invasive lobular carcinoma of the breast (4). Other rarer locations are the gallbladder, biliary tract, pancreas, small intestine, ampulla of Vater, cervix, and urinary bladder/urachus (2, 5, 6). It is hypothesized that KTs develop as a result of selective retrograde lymphatic dissemination of the primary gastric malignancy along the stomach-to-ovary lymphatic pathway (7).

These tumors tend to appear at a younger age, with a median age of onset in the fourth decade of life, notably earlier than most primary ovarian malignancies, which are usually diagnosed between the ages of 55 and 65 (2). The KTs diagnosis primarily relies on identifying their distinctive histopathological characteristics under microscopy, which typically include a dense fibroblastic stroma infiltrated by malignant signet ring cells arranged individually, in nests, or in cord-like patterns. Additionally, immunohistochemical (IHC) analysis plays a crucial role in differentiating between primary ovarian neoplasms and metastatic lesions (3).

KTs are linked to a negative outcome and are typically a sign of advanced-stage cancer. Due to the lack of a well-established effective treatment, the prognosis remains poor, with most patients reporting a median survival of about 14 months after diagnosis (7).

Although these tumors are mostly found in adult women, they are extremely uncommon in adolescents and young adults. Because of their unusual presentation in this age group, they frequently result in major delays in diagnosis. Given the limited number of reported cases in individuals under 20 years of age, we present a rare KT case in a teenage patient and complement this with a narrative review of the existing literature. Our aim is to offer a descriptive synthesis of published case reports, case series, and reviews of the literature focusing on this specific age group. This approach enables a better understanding of the clinical features, pathological findings, and management options relevant to this rare population.

## 2 Case description

### 2.1 Clinical and biological findings

An 18-year-old female was admitted to our clinic with a six-month history of progressive abdominal distension and intermittent lower abdominal pain, both of which had worsened in the preceding month. Prior to admission, the patient had sought care at multiple provincial medical centers, where non-specific diagnostic investigations were conducted. She was later admitted to a general surgery department, where imaging studies suggested the presence of a pelvic mass, and an exploratory laparotomy was advised. However, the procedure was declined by the patient’s mother due to concerns regarding the invasiveness and associated surgical risks. The patient was subsequently referred to our institution for further diagnostic evaluation and management.

The patient’s clinical history has been recorded and physical examination, including bimanual pelvic assessment was performed. Imaging was initiated with transabdominal ultrasonography to evaluate the pelvic masses, followed by serum biomarker testing. The patient’s medical history was unremarkable, with no known familial predisposition to malignancy. Menstrual cycles were regular, and no prior or current gynecologic symptoms were reported. On clinical examination, vital signs were within normal limits. Abdominal palpation revealed diffuse tenderness, and bimanual pelvic examination identified bilateral adnexal masses. Transabdominal ultrasonography demonstrated two large ovarian masses. The right ovary was entirely replaced by a predominantly solid, heterogeneous mass measuring 25 × 10 cm, while the left ovary exhibited a similar lesion approximately 10 cm in diameter. Serum tumor markers were within normal reference ranges, including alpha-fetoprotein (AFP: 0.944 IU/ml; normal: <10 IU/ml), cancer antigen 125 (CA-125: 9.18 IU/ml; normal: <35 IU/ml), and carcinoembryonic antigen (CEA: 1.34 ng/ml; normal: <5 ng/ml). Due to the emergency nature of the case, the serum levels of LDH, β-hCG, and CA 19–9 were not immediately available at the time of initial surgical management. These were obtained postoperatively and showed elevated values for CA 19-9 (66.83 IU/mL; normal range: < 15 IU/mL) and LDH (205 U/L; normal range: 135–225 U/L)while β-hCG was unavailable in emergency testing. Mild elevations were observed in platelet count and C-reactive protein (CRP), though exact values remained just above the upper normal limits.

Exploratory laparotomy with intraoperative frozen section analysis was initially performed based on sonographic findings and a previously obtained contrast-enhanced CT scan from another center, which described only bilateral pelvic masses without suspicion of a gastric origin. As the patient’s condition required urgent intervention, gastrointestinal endoscopy was not performed preoperatively. Based on intraoperative findings, patient age and subsequent histopathology, both upper GI endoscopy and updated imaging were conducted postoperatively to identify a potential primary malignancy. These included upper gastrointestinal endoscopy and colonoscopy, with targeted biopsy collection. Cross-sectional imaging with contrast-enhanced computed tomography (CT) of the head, chest, abdomen, and pelvis was also performed to assess disease extent and identify any primary lesions. Histopathological and immunohistochemical analyses were subsequently carried out on both gynecologic and gastrointestinal specimens.

### 2.2 Endoscopic and medical imaging assessments

#### 2.2.1 Contrast computed tomography

**Figure 1:**
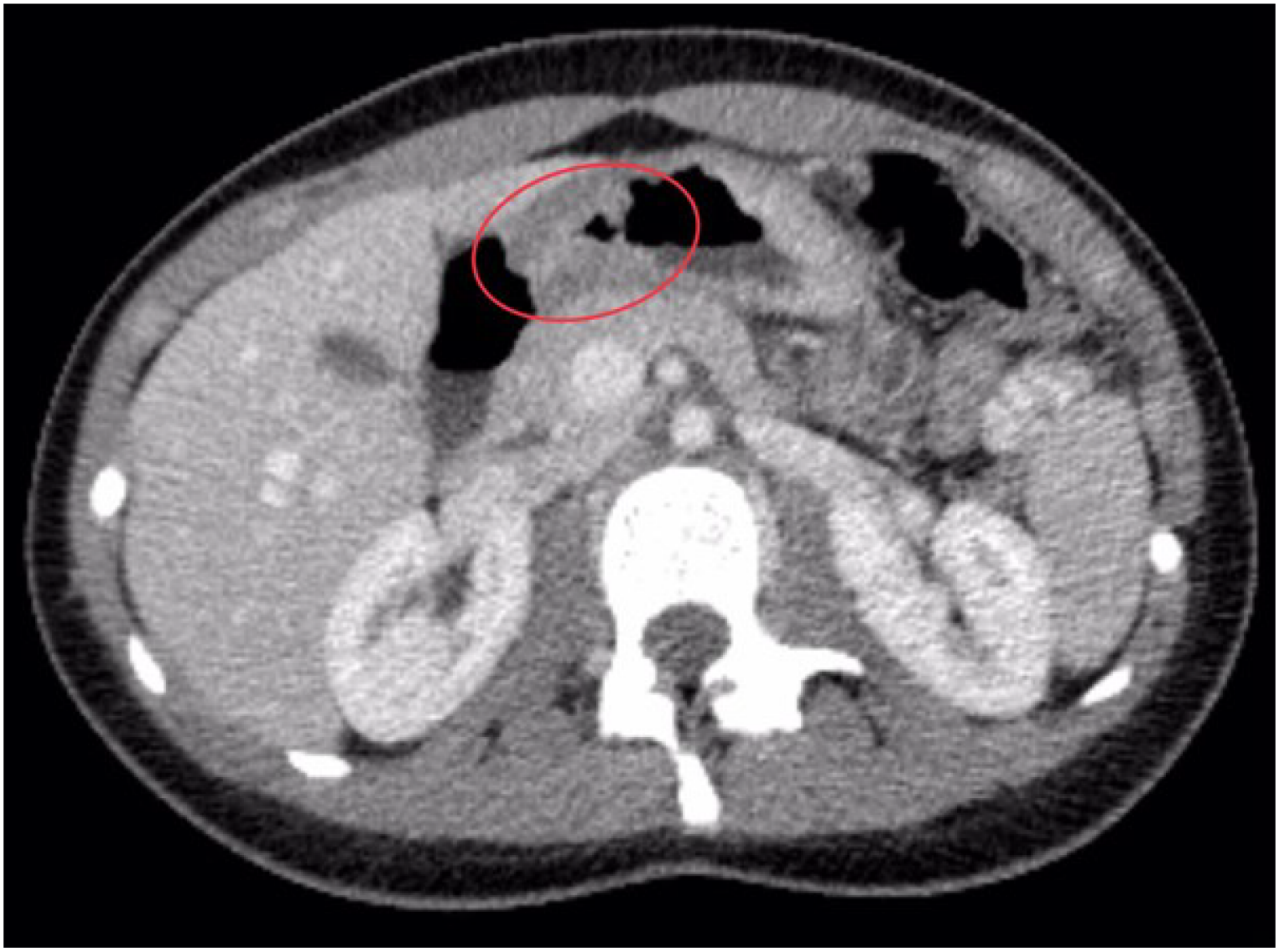
Axial contrast-enhanced CT image (portal venous phase) showing circumferential wall thickening of the gastric antrum extending into the pyloric region. The gastric wall demonstrates uniform thickening measuring approximately 9 mm (see red oval), associated with luminal narrowing and reduced contrast enhancement (iodophilic pattern). No signs of perigastric fat stranding or adjacent organ invasion are observed at this level. The radiological appearance raised strong suspicion for a primary gastric malignancy.

**Figure 2:**
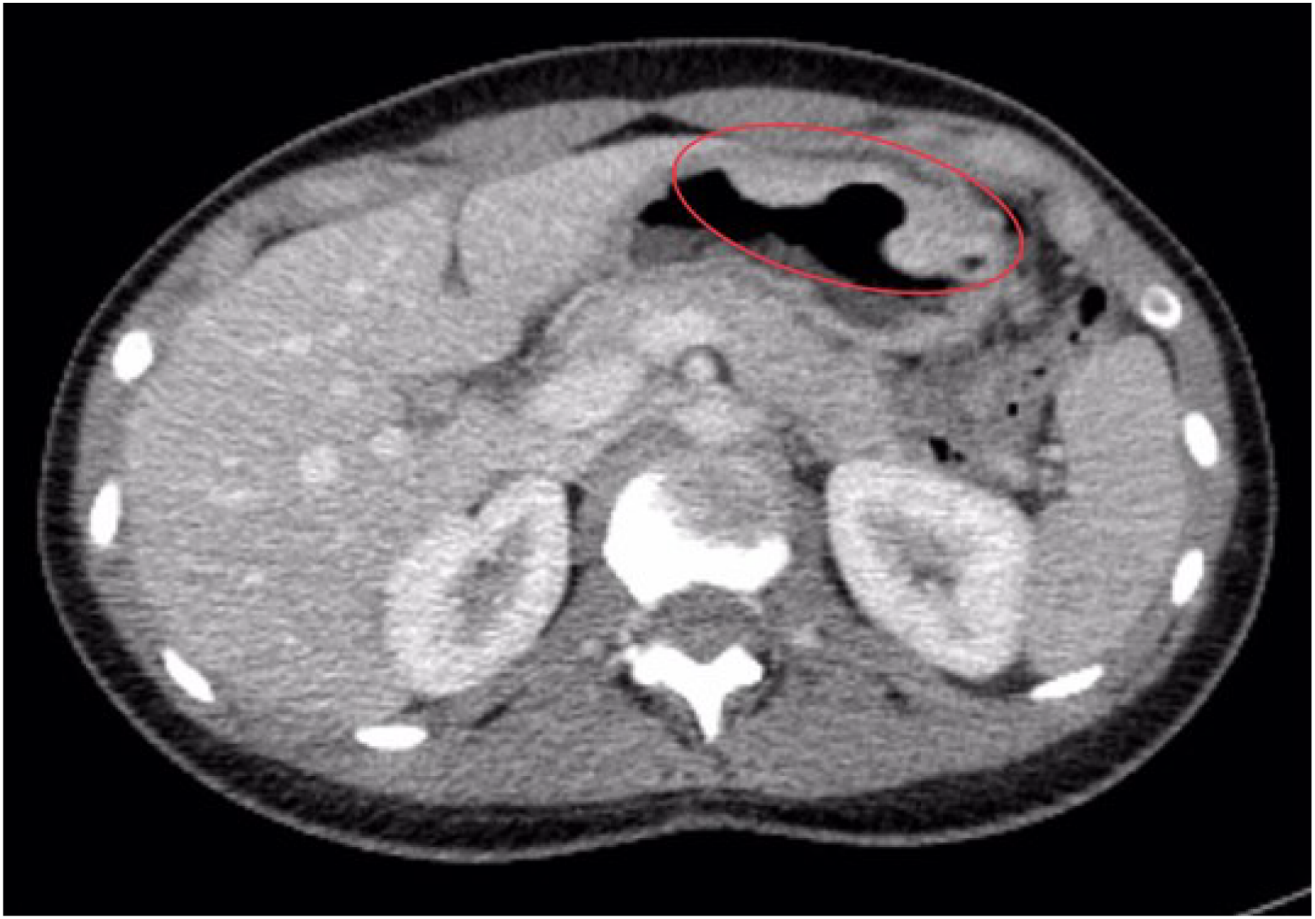
Axial contrast-enhanced CT scan (portal venous phase) showing focal nodular thickening along the gastric greater curvature. The lesion measures approximately 11 mm in thickness, with a focally exophytic contour and mild iodophilia. Its bosselated, endoluminally protruding appearance raised suspicion for a primary gastric malignancy.

To assess the extent of disease dissemination and to identify a potential primary malignancy, a contrast-enhanced computed tomography (CT) scan of the head, chest, abdomen, and pelvis was conducted. The liver appeared enlarged and mildly lobulated, although no definitive focal hepatic lesions were identified. The left adnexa was noted to be enlarged due to a hypodense mass, which was clearly visible on both non-contrast and contrast-enhanced sequences. Additionally, lymphadenopathy was observed in the left external iliac region, with the largest lymph node measuring approximately 13 × 14 mm. At the level of the gastric antrum, the CT scan revealed circumferential wall thickening measuring 9 mm, resulting in narrowing of the gastropyloric junction (**Figure 1**), with minimal contrast enhancement (iodophilia). Moreover, a focally exophytic, iodophilic thickening measuring 11 mm was detected along the greater curvature of the stomach, exhibiting an endoluminally protruding, bosselated contour (**Figure 2**), findings that were highly suggestive of a primary gastric malignancy.

#### 2.2.2 Inferior endoscopy

Considering the patient’s young age and the atypical macroscopic appearance of the adnexal lesions, additional diagnostic work-up for a potential gastrointestinal primary malignancy was initiated two weeks postoperatively. The patient underwent both upper gastrointestinal endoscopy and colonoscopy. The colonoscopic examination revealed only small internal hemorrhoids, with no signs of neoplastic involvement.

**Figure 3:**
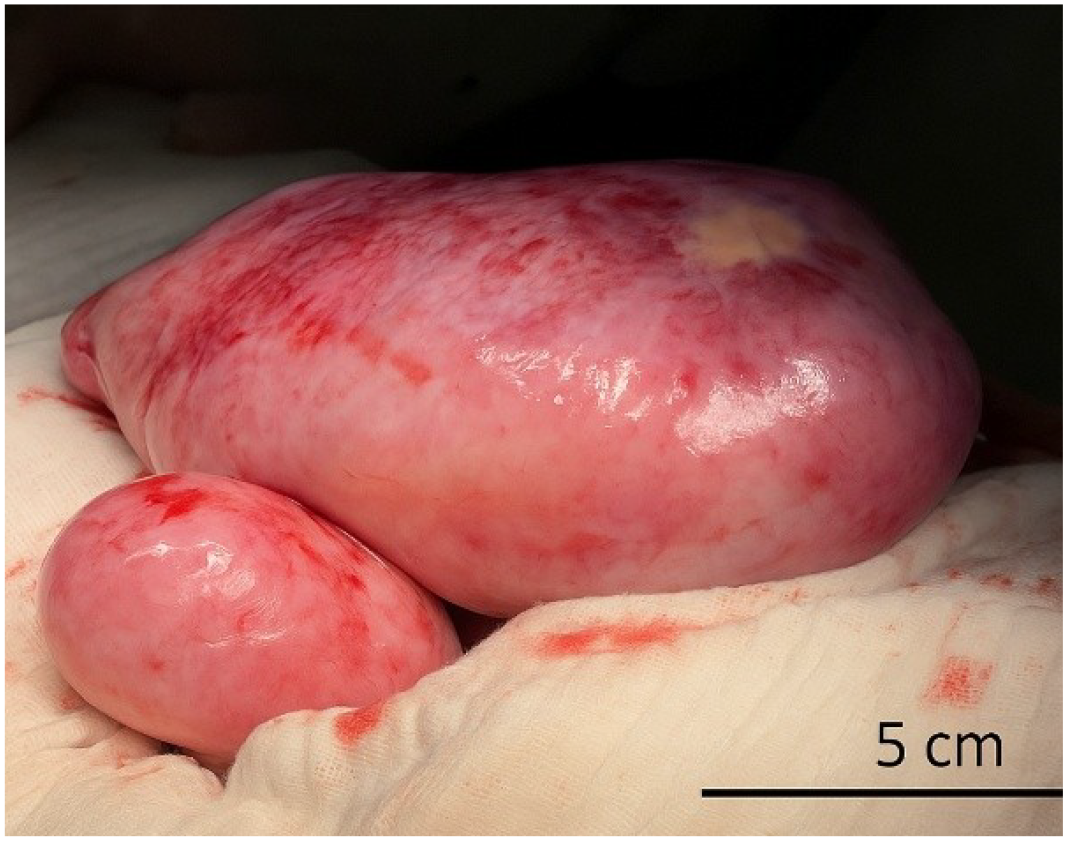
Intraoperative photograph showing bilateral ovarian tumor masses. The right ovary (upper mass) is markedly enlarged and entirely replaced by a lobulated, smooth-surfaced tumor measuring approximately 25 × 10 cm, with serosal congestion and areas of focal discoloration. The left ovary (lower mass) also exhibits tumoral transformation, measuring approximately 10 × 8 cm. No surface rupture or ascites was noted at the time of exploration.

Conversely, upper gastrointestinal endoscopy identified a large, suspicious ulcer located at the gastric angle, characterized by a necrotic, debris-covered crater with irregular margins, raising concern for an underlying malignant process (**Figure 3**). Biopsy samples were obtained from both the base and the edges of the ulcer for histopathological examination.

### 2.3 Surgical treatment and tumor macroscopic features

**Figure 4:**
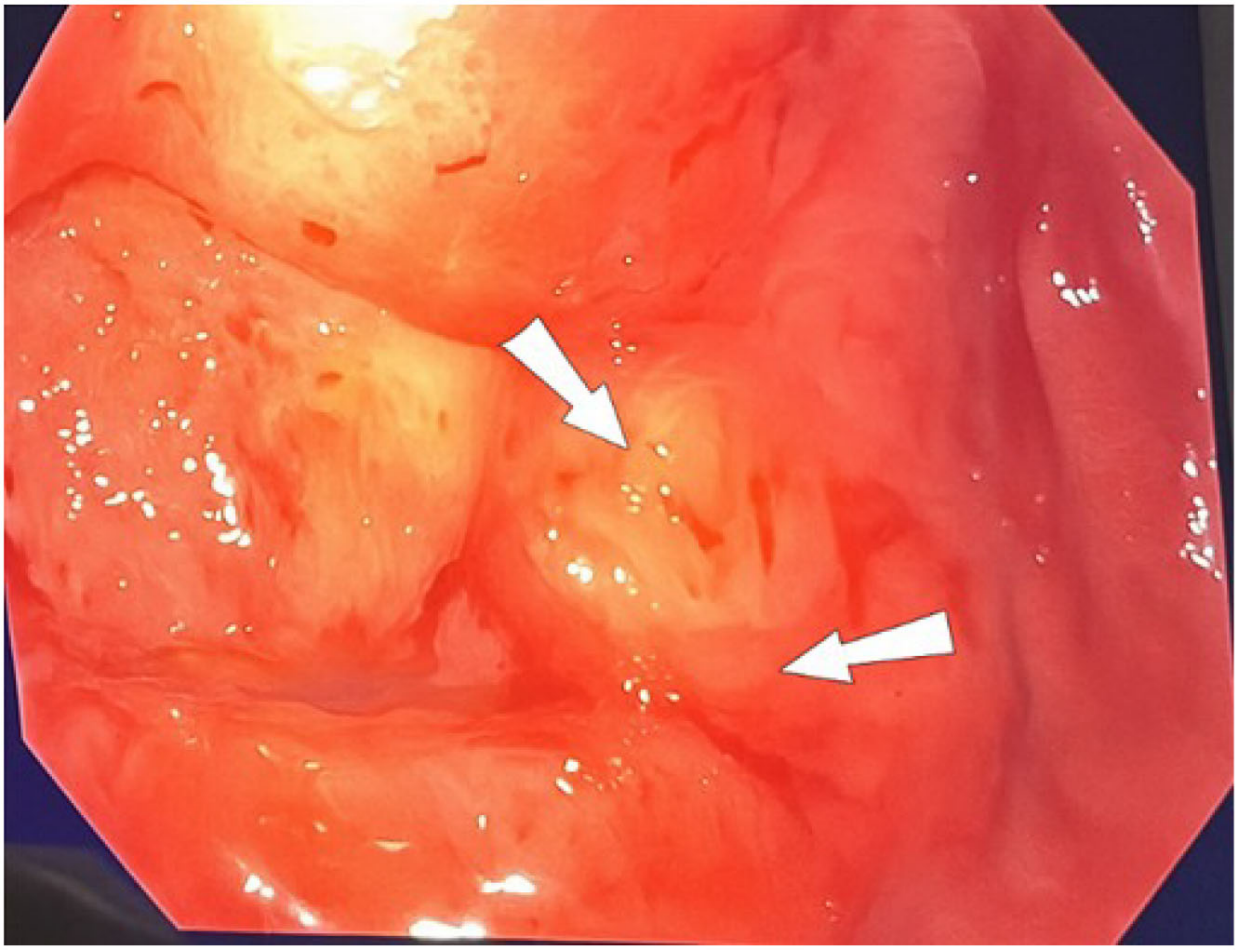
Endoscopic view of the ulcerated gastric mass. Upper gastrointestinal endoscopy revealed a large ulcerative lesion located at the gastric angle. The lesion exhibited a necrotic base, irregular elevated borders (arrows), mucosal friability, and no signs of active bleeding. These features raised suspicion for an underlying poorly cohesive carcinoma. Biopsies were taken from the base and edges of the lesion for histopathological confirmation.

The patient was scheduled for surgical intervention, and an exploratory laparotomy was performed. Intraoperatively, the right adnexa was found to be completely replaced by a tumor mass measuring approximately 25 × 10 cm, with irregular margins and a heterogeneous consistency (**Figure 4**). Notably, the mass exhibited two complete torsions around its vascular pedicle. Manipulation of the lesion led to the release of serous, citrine-colored fluid.

The left ovary also demonstrated tumoral characteristics, measuring 10 × 8 cm, and exhibited a firm consistency on palpation. Additionally, a 4 × 2 cm nodular lesion was observed on the peritoneal surface. A biopsy specimen was obtained from this lesion for histopathological analysis. No ascites was present at the time of surgery. Surgical management consisted of a right and left adnexectomies, with the specimen submitted for intraoperative frozen section analysis, during which a strip of grossly unremarkable ovarian tissue was preserved with the intent of maintaining residual endocrine function. Frozen section analysis of the right adnexal mass raised suspicion for a sex cord-stromal tumor; however, the possibility of a secondary metastatic lesion could not be definitively excluded intraoperatively.

### 2.4 Pathology and immunochemistry

**Figure 5:**
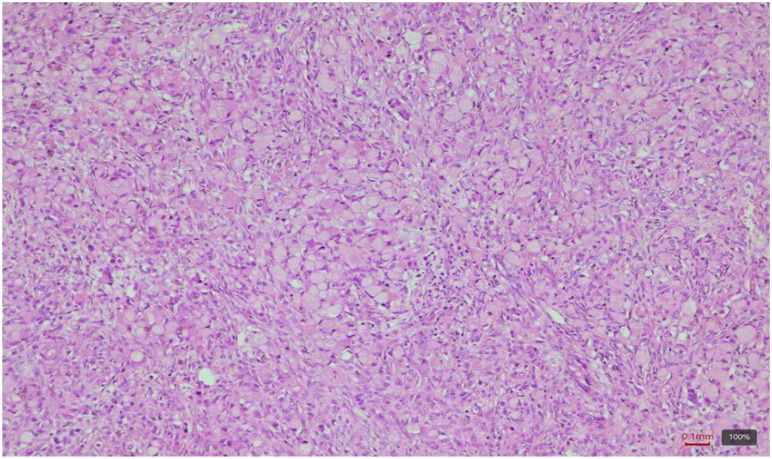
Ovarian parenchyma with diffuse tumoral infiltration with signet ring cells. HE, ob. 10x.

The histopathological examination in the usual hematoxylin-eosin staining revealed ovarian parenchyma with architecture completely replaced by a tumoral infiltration composed of isolated or grouped cells with plasmacytoid morphology and signet ring (**Figure 5**). The special PAS (periodic acid-schiff) staining was positive and revealed the presence of neutral mucins in the cytoplasm of the tumoral cells. Considering the morphological peculiarity of the tumoral cells, the differential diagnosis between a stromal tumor with cells with “signet ring-like” morphology and a metastasis from a carcinoma of gastrointestinal origin was taken into account.

Based on these results, the patient was referred to the oncology department and initiated on systemic chemotherapy. Unfortunately, postoperative follow-up was limited due to poor compliance with recommended surveillance and treatment protocols by the patient’s family, thereby impeding accurate long-term outcome assessment.

**Figure 6:**
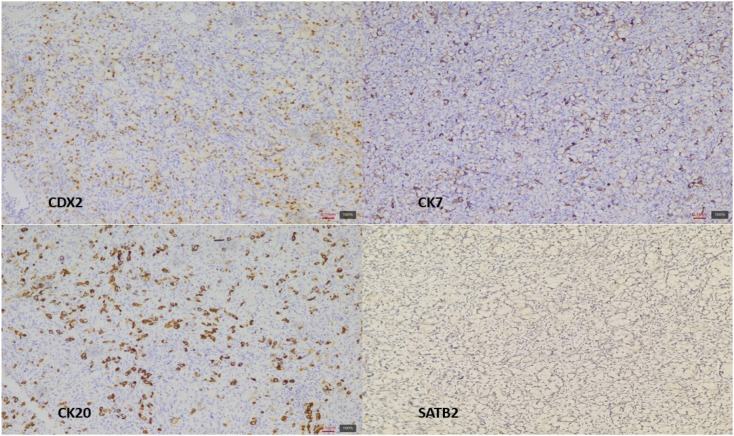
Tumor immunohistochemical profile. Note nuclear positivity for CDX2, along with membrane positivity for CK20 and CK7. SATB2 without nuclear expression in the tumor.

Immunohistochemical (IHC) tests revealed an epithelial tumor (AE1/AE3 positive staining) with an immunohistochemical profile CK20+/CK7+/CDX2+/SATB2- (**Figure 6**). Stromal tumor with cells with “signet ring-like” morphology was excluded by AE1/AE3 positivity and negative inhibin, directing the diagnosis towards a secondary determination of a signet ring cell carcinoma of gastrointestinal origin, most likely gastric.

**Figure 7:**
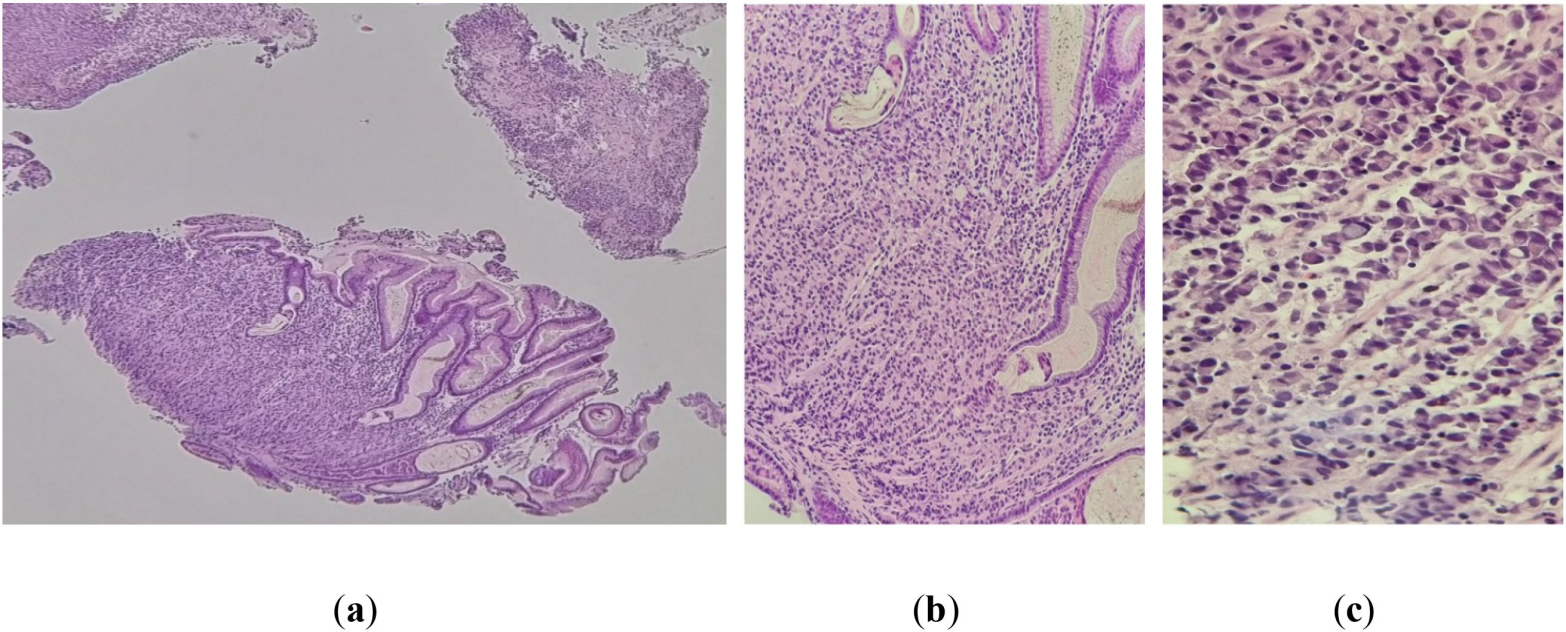
**(a)** Gastric mucosa with partially remodeled architecture by tumor infiltration. HE, ob. 4x. **(b)** Detailed image of the gastric mucosa. HE, ob. 10x. **(c)** Note the tumor infiltration with cells with signet ring morphology. HE, ob.20x.

Biopsy fragments taken from the ulcerated gastric tumor formation reveal focal mucosal architecture (**Figure 7A**) replaced by a tumor proliferation composed of cords of discohesive cells, most of which present signet ring cell morphology (**Figures 7B, C**).

## 3 Discussion

### 3.1 Literature research methodology

This study was conducted as a narrative literature review, given the exceptional rarity of KT cases reported in adolescents and young adults. The primary objective was to identify relevant case reports, case series, and review articles, and to synthesize data regarding the clinical presentation, pathological features, and therapeutic approaches associated with KTs diagnosed in patients under the age of 20.

A comprehensive search of the literature was carried out up to March 2025 across three electronic databases: PubMed, Google Scholar, and ScienceDirect. The search strategy employed combinations of the following keywords: “young age,” “ovarian metastasis,” “teenage,” “Krukenberg tumor,” “metastatic tumor,” and “signet-ring cell.” Only articles published in English were considered, and the selection was limited to case reports and case series.

Titles and abstracts of all identified records were screened, and full-text articles were retrieved for those meeting eligibility criteria. Preference was given to peer-reviewed publications that included detailed clinical, histopathological, or immunohistochemical data. The inclusion criteria were as follows:

- Articles published in peer-reviewed journals;
- Case reports or case series involving patients under 20 years of age with a confirmed diagnosis of Krukenberg tumor;
- Studies providing information on histopathology, immunohistochemistry, or clinical outcomes;
- Meta-analyses and systematic reviews focused on the epidemiology, diagnostic strategies, or prognosis of Krukenberg tumors;
- English-language publications.
- Exclusion criteria included studies lacking patient-specific data or those unrelated to the defined age group.

These sources were used to contextualize the present case and to highlight patterns, similarities, and variations in clinical presentation, tumor origin, bilateral ovarian involvement, and prognosis among patients within this age group. Given the scarcity and heterogeneity of reported cases, a formal meta-analysis was not feasible. Consequently, the present review offers a descriptive and contextual synthesis of the available literature.

### 3.2 Cases of young patients (under 20 years old) found in literature

To better contextualize our case within the available literature, we performed a narrative review of previously documented KTs in patients less than 20 years old. A total of 15 articles were selected based on their clinical and histopathological relevance. These included individual case reports in patients aged 11, 13, 15, 16, 18, and 20 years, as well as broader series and meta-analyses providing statistical insights into the presentation and KTs outcomes. The reviewed literature consisted of peer-reviewed case reports (3, 5–9), systematic reviews and meta-analyses (4, 10), narrative or pathological overviews (2, 11–13), and clinical or retrospective studies (1, 4, 14).

Ruotolo et al. (7) described a case of an 11-year-old girl who presented with abdominal discomfort and distension; intraoperative frozen section analysis revealed bilateral KTs originating from a gastric adenocarcinoma, which was confirmed via endoscopy and biopsy. Similarly, Khurana et al. (8) documented a 13-year-old patient with abdominal enlargement, in whom the diagnosis was made postoperatively through histopathological analysis.

The case reported by Sahin et al. (9) describe a 15-year-old girl who was diagnosed with metastatic colon adenocarcinoma manifesting as bilateral Krukenberg tumors. Similar to our case, the patient describes nonspecific abdominal symptoms and was initially diagnosed based on adnexal imaging.

Annal and Sadath (5) presented the case of a 16-year-old girl who initially reported irregular menstruation and lower abdominal pain; intraoperative frozen section and upper GI endoscopy confirmed bilateral ovarian metastases from a gastric primary tumor, similar presentation in an 18-year-old patient (6) with non-specific pelvic symptoms and an eventual KT diagnosis following frozen section biopsy and gastric endoscopy.

According to Parmar et al. (3), a 20-year-old woman presented with abdominal bloating and pain, later diagnosed with bilateral KTs confirmed through CT imaging and histopathology.

The selected case reports, presented in **Table 1**, highlight consistent clinical features such as bilateral ovarian involvement, nonspecific abdominal symptoms, and a gastric primary tumor, often of signet-ring cell type. All six cases shared characteristics of nonspecific abdominal symptoms, such as abdominal discomfort or distension, also all six cases present bilateral ovarian involvement and a gastrointestinal primary tumor, an intraoperative diagnosis being established in three of them. Despite the heterogeneity of symptoms in the initial presentation, diagnostic approaches often included a combination of imaging, endoscopic evaluation, and histopathological confirmation.

**Table 1:** Summary of published case reports on KTs in young patients (≤20 years).

First author (year)	Age	Symptoms	Ovarian involvement	Primary tumor site	Diagnostic methods	Treatment	Outcome
Ruotolo et al. (7)	11	Abdominal pain, distension, swellling	Bilateral	Unknown	US, CT, Endoscopy, Biopsy	Bilateral oophorectomy, chemotherapy	Poor, rapid progression- death
Khurana et al. (2010) (8)	13	Abdominal mass, nausea	Bilateral	Sigmoid colon	US, Histology	Surgery	Not specified
Sahin et al. (2019) (9)	15	Abdominal pain, distension	Bilateral	Colon (signet-ring)	US, MRI, histopathology, PET-CT	Surgery, chemotherapy, HIPEC	Partial response, then progression
Kuno et al. (2016) (6)	18	Pelvic pain	Bilateral	Transverse colon	CT, MRI Endoscopy, Histopathology	Surgery, chemotherapy	Not specified
Annal & Sadath (2022) (5)	16	Amenorrhea, pain	Bilateral	Gastric	Imaging, biopsy	Surgery	Rapid progression- death
Parmar et al. (2021) (3)	20	Pelvic pain, weight loss	Bilateral	Gastric	CT, Endoscopy, IHC	Surgery, chemotherapy	Alive, under follow-up

CT, computer tomography; HIPEC, hyperthermic intraperitoneal chemotherapy; IHC, immunohistochemistry, MRI, magnetic resonance imaging, PET-CT, positron emission computed tomography, US-ultrasound.

Notably, treatment options varied according to disease stage and institutional resources. In most cases management generally consist of a multimodal approach including surgical resection and systemic chemotherapy. The treatment techniques can vary depending on clinical presentation and stage of diagnosis. Surgery was employed, typically through bilateral salpingo-oophorectomy, sometimes with additional removal of visible metastases. While the majority of patients underwent surgical removal of the ovarian tumors, systemic chemotherapy was inconsistently used. In most cases, treatment consisted of surgical intervention, often followed by chemotherapy, with overall poor prognostic outcomes in most cases. One case (the 20-year-old patient (3)) also received palliative care due to advanced disease progression at the time of diagnosis.

This comparison highlights the diagnostic complexity and clinical challenges associated with early identification and KTs management in adolescents and young adults. These cases underscore the importance of considering metastatic disease in the differential diagnosis of adnexal masses in young females, particularly when accompanied by gastrointestinal symptoms or abnormal menstrual history. The accompanying literature review attempts to contextualize the rarity and challenges of this diagnosis, advocating for the inclusion of gastrointestinal sources in the differential diagnosis of ovarian masses in young females.

### 3.3 Tumor features within old and new definition

In the present case, bilateral ovarian masses in an 18-year-old patient were ultimately confirmed to be metastatic signet-ring cell carcinoma of gastric origin, consistent with the classical definition of Krukenberg tumors. This diagnosis aligns with the histopathological hallmark first described by Friedrich Krukenberg in 1896, who characterized these tumors by the presence of mucin-filled signet-ring cells and a prominent proliferation of the ovarian stroma (2). The World Health Organization (WHO) diagnostic criteria further specify that KTs must exhibit stromal involvement, mucin-producing neoplastic signet-ring cells, and sarcomatoid proliferation of the ovarian stroma (2, 3).

Although originally defined with strict morphological parameters, in clinical practice the term “Krukenberg tumor” is often applied more broadly to any metastatic ovarian carcinoma, most frequently originating from gastrointestinal malignancies such as gastric adenocarcinomas (2, 3). The features observed in our patient therefore represent both the historical and modern conceptual framework of Krukenberg tumors, demonstrating how these criteria remain clinically relevant even in rare cases occurring in adolescents.

### 3.4 Case rarity as per current epidemiology

The bilateral ovarian metastases in this casa originate from a primary gastric signet-ring cell carcinoma, which is an extreme rarity of KTs in adolescents. Most reported KTs arise from primary gastrointestinal malignancies, with gastric adenocarcinomas representing the predominant source (2). While the stomach remains the most frequent primary site (70% of cases) (2, 3, 5, 7), some recent studies report a higher relative incidence of colorectal primaries (45.31%) compared with gastric (32.03%) (4).

Gastric carcinoma often remains asymptomatic until advanced stages, which necessitates maintaining a high index of suspicion and performing early upper gastrointestinal endoscopy, even in young individuals (4). Primary ovarian mucinous carcinomas are themselves uncommon, accounting for only 3–5% of all epithelial ovarian cancers; a considerable proportion of ovarian mucinous tumors previously thought to be primary are now recognized as metastatic, most frequently from the gastrointestinal tract.

In one of the largest series comprising 120 KT cases, only four patients (3%) were identified between the ages of 10 and 20, including a 13-year-old adolescent (12), highlighting the exceptional rarity of this condition in pediatric and teenage populations. Our literature review identified six additional cases in patients younger than 20 years, most of which were associated with a gastric primary (3, 5–9).

### 3.5 Non-specific clinical findings should not exclude rare type tumors

In the present case, the clinical presentation was progressive abdominal distension and intermittent lower abdominal pain, which had worsened over the preceding month. Bilateral adnexal masses identified during physical exam were revealed by imaging to be solid ovarian tumors. Thus, the case has a particular diagnostic challenge, since initial presentation could correspond to a primary ovarian malignancy, and symptoms are often nonspecific.

However, the findings in the clinical examination are consistent with the literature, where abdominal pain, abdominal distension, ascites, and a palpable pelvic mass are among the most frequently reported symptoms of KTs (2, 4). The results of a 2020 study published in the *World Journal of Clinical Cases* (10) meticulously analyzed 48 individual studies and included a total of 3,025 patients diagnosed with Krukenberg tumors. This study shows that 64.3% of cases exhibited bilateral ovarian involvement, and common presenting symptoms included ascites (51.7%), palpable mass (31.3%), abdominal pain (29.3%), and abdominal distension (28.7%). In rare cases, KTs have been identified during pregnancy, eight patients were discovered to be pregnant at the time of diagnosis, with two of the ovarian tumors being incidental findings during delivery (13). Other, less common manifestations include virilization, attributed to hormone production by the ovarian stroma under human chorionic gonadotropin (HCG) stimulation, particularly during pregnancy (2, 3, 12, 13), and abnormal vaginal bleeding (12).

This case highlights the importance of maintaining a high index of suspicion for this rare condition in younger patients, especially when ovarian masses are accompanied by vague gastrointestinal or gynecologic symptoms. All six cases of young patients shared characteristics of nonspecific abdominal symptoms, such as abdominal discomfort or distension, also all six cases present bilateral ovarian involvement and a gastrointestinal primary tumor, an intraoperative diagnosis being established in three of them.

Importantly, KTs being extremely uncommon in adolescents and young adults often cause significant diagnostic delays due to their unusual occurrence in this age group and the nonspecific nature of early symptoms (3, 5–8, 12, 15).

Furthermore, radiological findings are typical i.e. bilateral, solid ovarian masses, although cystic forms are also described (2, 3, 6, 12), as observed in our case, where both ovaries were replaced by large tumoral masses. Furthermore, the primary gastrointestinal tumor is often small or clinically occult at the time of ovarian tumor detection (2, 3, 5, 7, 12), and a history of a known primary carcinoma is obtained in only 20–30% of patients (2).

Laboratory evaluation showed serum tumor markers within normal limits, including CA 125, which was not elevated preoperatively. However serum CA 125 levels can be elevated (1–3, 5, 14) and can be used for postoperative monitoring and to estimate prognosis (a lower 5-year survival rate in patients with preoperative CA 125 levels > 75 U/mL) (2).

### 3.6 Pathology and immunochemistry leading to defintitive diagnostic

In the present case, both ovaries were enlarged, with the right adnexa completely replaced by a solid, heterogeneous mass and the left ovary demonstrating a firm tumoral lesion. The external surfaces were smooth, without signs of rupture or ascites, and a peritoneal nodular lesion was separately identified. These findings are consistent with the classical pathological features described for Krukenberg tumors, which are bilateral in more than 80% of reported cases (2, 3, 5, 12, 15), often asymmetrically enlarged and exhibiting a bosselated contour (2, 3, 8, 12). On sectioning, KTs typically present yellow or white cut surfaces, most commonly solid but occasionally cystic (2, 3, 12). Another distinctive feature is their smooth capsular surface, generally free of adhesions or diffuse peritoneal deposits (2, 3, 5, 12) a characteristic also observed in our patient.

In our patient, the ovarian parenchyma was completely replaced by tumor infiltration composed of isolated and grouped cells showing plasmacytoid morphology and classic signet-ring features, confirmed by positive PAS staining for neutral mucins. IHC revealed an epithelial tumor (AE1/AE3 positive) with a CK20+/CK7+/CDX2+ profile, supporting its metastatic origin from a gastrointestinal primary rather than a primary ovarian neoplasm. These findings are consistent with the known histological hallmarks of Krukenberg tumors, where the epithelial component is predominantly composed of mucin-laden signet-ring cells with eccentric hyperchromatic nuclei (2, 3, 5, 8, 12). These cells may be arranged individually, in clusters, nests, cords, trabeculae, acini, or tubules, and a tubular variant of KT has also been described (2, 8, 12). The mesenchymal (stromal) component, typically of ovarian stromal origin, consists of plump and spindle-shaped cells with minimal cytological atypia and limited mitotic activity (1, 6). In some cases, the stroma may demonstrate edema, pseudocyst formation, or a pronounced desmoplastic reaction that can obscure the signet-ring cells (2, 12).

Also, one of another crucial diagnostic challenges is distinguishing primary ovarian mucinous adenocarcinomas from metastatic mucinous adenocarcinomas involving the ovaries. Histochemical techniques such as Mayer mucicarmine, Periodic Acid–Schiff (PAS) with diastase digestion, and Alcian blue staining remain essential for confirming intracytoplasmic mucin (4–6, 8). In our case, histopathological analysis revealed a complete replacement of the ovarian parenchyma by tumor infiltration composed of isolated and grouped cells with signet-ring morphology, accompanied by abundant intracytoplasmic mucin. This morphological pattern is strongly indicative of a metastatic rather than a primary ovarian tumor, as signet-ring cells are rarely observed in primary ovarian mucinous carcinomas (3, 6, 8, 15). Special PAS staining confirmed the presence of neutral mucins within the tumor cells, further supporting a metastatic mucinous carcinoma phenotype.

Markers such as CK7, CK20, CDX2, PAX8, SATB2, and MUC family proteins are helpful in immunochemistry panels to guide diagnosis. For example, gastrointestinal primaries (especially colorectal) are often CK20+, CDX2+, and PAX8–, while primary ovarian tumors are typically CK7+, PAX8+, and CDX2– (3, 11). IHC profiling in the present case demonstrated an epithelial origin (AE1/AE3 positivity) with a CK20+/CK7+/CDX2+/SATB2− profile, while inhibin staining was negative, effectively excluding a primary sex cord-stromal tumor. The immunoprofile was compatible with a gastrointestinal primary, most likely gastric, aligning with the ulcerated gastric lesion confirmed endoscopically and histologically in this patient. Tumor cells are immunoreactive to epithelial markers (e.g., cytokeratins, EMA) and negative for vimentin and inhibin (2, 5). IHC (CK7 and CK20) aids in differentiating primary ovarian from metastatic lesions and in identifying the primary site (2, 3, 6, 12). For example, CK20 positive and CK7 negative favors colorectal origin (2, 3, 6, 15).

Our patient had no evidence of peritoneal carcinomatosis or capsular rupture during surgery which is consistent with the metastatic pathway described for KTs (2, 3, 5, 12). The main hypothesis explaining this pattern is selective retrograde lymphatic dissemination. This is supported by several observations: the frequent identification of lymphatic permeation at the ovarian hilum and cortex; the occurrence of KTs even in early gastric carcinomas limited to the mucosa and submucosa, which possess a dense lymphatic plexus; and the increased risk of ovarian metastases associated with higher numbers of metastatic lymph nodes (2, 3, 14). Previous assumptions suggested that premenopausal ovarian hormonal activity and rich vascularization might predispose to metastasis; however, Qiu et al. found no significant correlation between menopausal status and ovarian metastatic involvement (14). Our case, involving an adolescent patient, supports the concept that ovarian metastasis may occur independently of hormonal status, further emphasizing the importance of lymphatic spread in the pathogenesis of Krukenberg tumors.

### 3.7 Prognosis and prognostic factors

According to literature, the bilateral ovarian metastases from a poorly differentiated gastric signet-ring cell carcinoma are generally associated with an unfavorable outcome. In the reviewed cases, bilateral ovarian involvement was common, as was the rapid clinical development, underscoring the aggressive nature of signet-ring cell carcinomas in this age group (1, 4, 14).

KTs typically have a poor prognosis, with a median survival rate of approximately 14 months following diagnosis (2, 4, 5, 7, 15). Prognostic outcomes are strongly influenced by several factors. Among these, the depth of invasion of the primary gastrointestinal tumor (T stage) is considered the most critical predictor, with deeper invasion associated with earlier and more aggressive metastatic spread (14). The presence of ascites is an additional negative prognostic factor (4, 14), although it was absent in our patient at the time of surgical exploration, representing a potentially favorable sign.

Survival also varies depending on the primary tumor site, being longest for breast primaries (median survival of 31 months), intermediate for colorectal origins (21.5 months), and shortest for gastric primaries (11 months) (2, 4). Furthermore, synchronous metastases, such as in this case, are associated with shorter survival compared to metachronous presentations (2, 4). Another important factor is the completeness of cytoreductive surgery, with the absence of residual metastatic lesions conferring a more favorable prognosis (1). For example, Parmar et al. (2021) reported positive short-term outcomes after following cytoreductive surgery and adjuvant chemotherapy, but long-term prognosis remained poor in several cases due to advanced disease at diagnosis (3).

### 3.8 Management strategies

Our patient underwent exploratory laparotomy with bilateral adnexectomy and peritoneal biopsy, followed by referral for systemic chemotherapy. This management approach reflects the current challenge in treating Krukenberg tumors, as no well-established effective treatment protocol exists (2, 4, 5). The role of metastasectomy remains controversial, yet it has been identified as a potential positive prognostic factor in some series (1, 4). In addition, some authors have considered prophylactic bilateral oophorectomy during primary tumor surgery as a preventive measure, although this approach requires further validation (1, 2).When metastatic disease is confined to the ovaries, optimal cytoreductive surgery combined with aggressive systemic chemotherapy has been associated with improved survival outcomes (1, 2, 4, 15).

The distinction between the two tumor types is essential, as it significantly impacts treatment choices, including chemotherapy selection, surgical planning, and the overall patient prognosis (8).Various chemotherapy regimens, including platinum-based combinations, 5-fluorouracil, oxaliplatin, docetaxel, and paclitaxel, have shown survival benefits, particularly when aggressive treatment schedules are applied (1, 4, 15). Despite partial radiologic response to FOLFOXIRI and Bevacizumab, disease progression occurred, requiring additional cytoreductive surgery and HIPEC (15).

Early diagnosis and prompt initiation of treatment are essential, especially in young patients, where diagnostic delays are frequent and often lead to advanced disease at presentation (2, 8, 15). The implementation of national registries is recommended to facilitate systematic data collection, improve diagnostic accuracy, and guide treatment strategies (2, 4). Moreover, gastrointestinal endoscopy should be considered routinely in patients with ovarian tumors of uncertain origin to enable timely detection of occult primary malignancies (2–5).
